# Supplementary material for: Microbial Community in the Permafrost Thaw Gradient in the South of the Vitim Plateau (Buryatia, Russia)
Source: Microorganisms. 2022 Nov 7;10(11):2202. doi: 10.3390/microorganisms10112202 (PMC9695985; doi:10.3390/microorganisms10112202)
Supplement: Supplementary file 1 [file microorganisms-10-02202-s001.zip › microorganisms-1969863-supplementary.pdf]

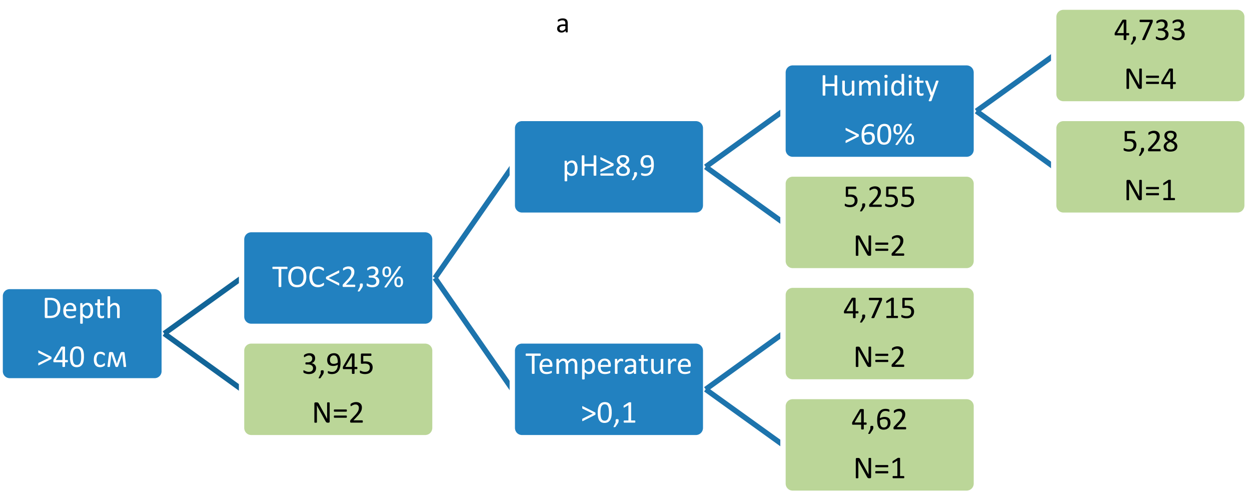

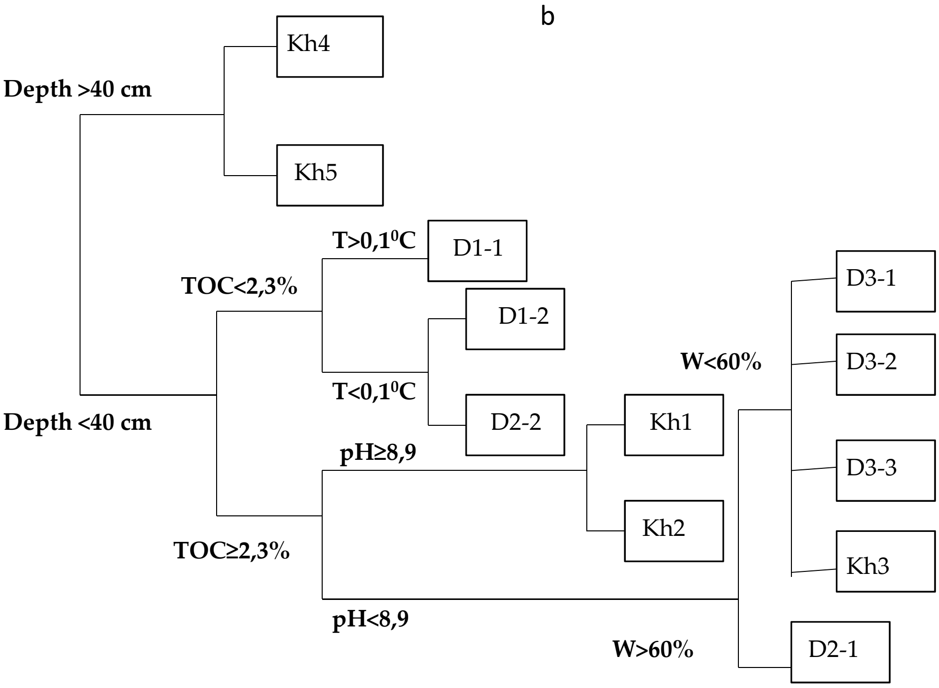

Figure S1 Regression tree analysis for assessing the effects of each environmental variables (a) and sample sites clustering (b). TOC – total organic carbon, T – soil temperature, W – soil humidity.

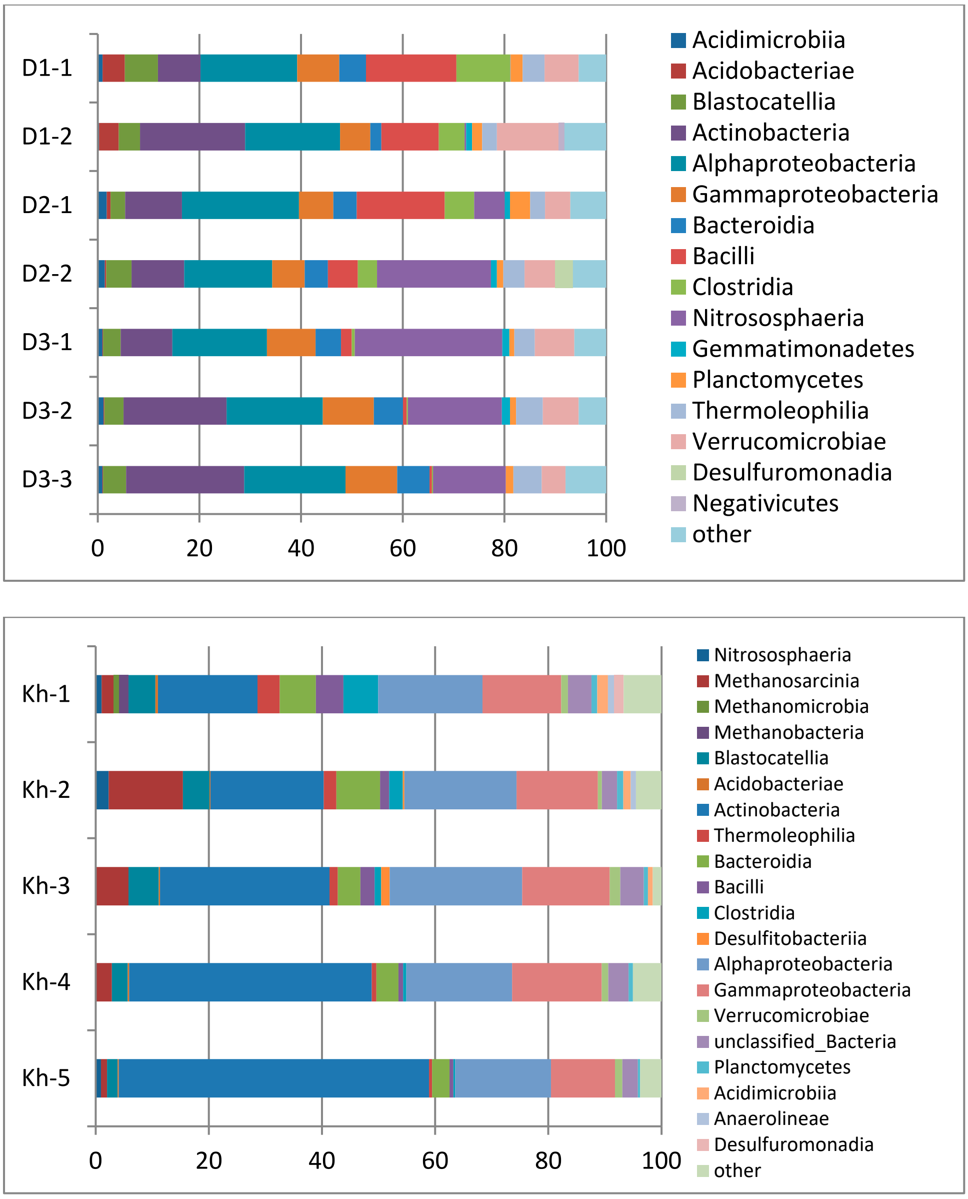

Figure S2 Relative abundance of bacteria and archaea on the class level in permafrost soils along Darkhituy-Khaimisan transect.

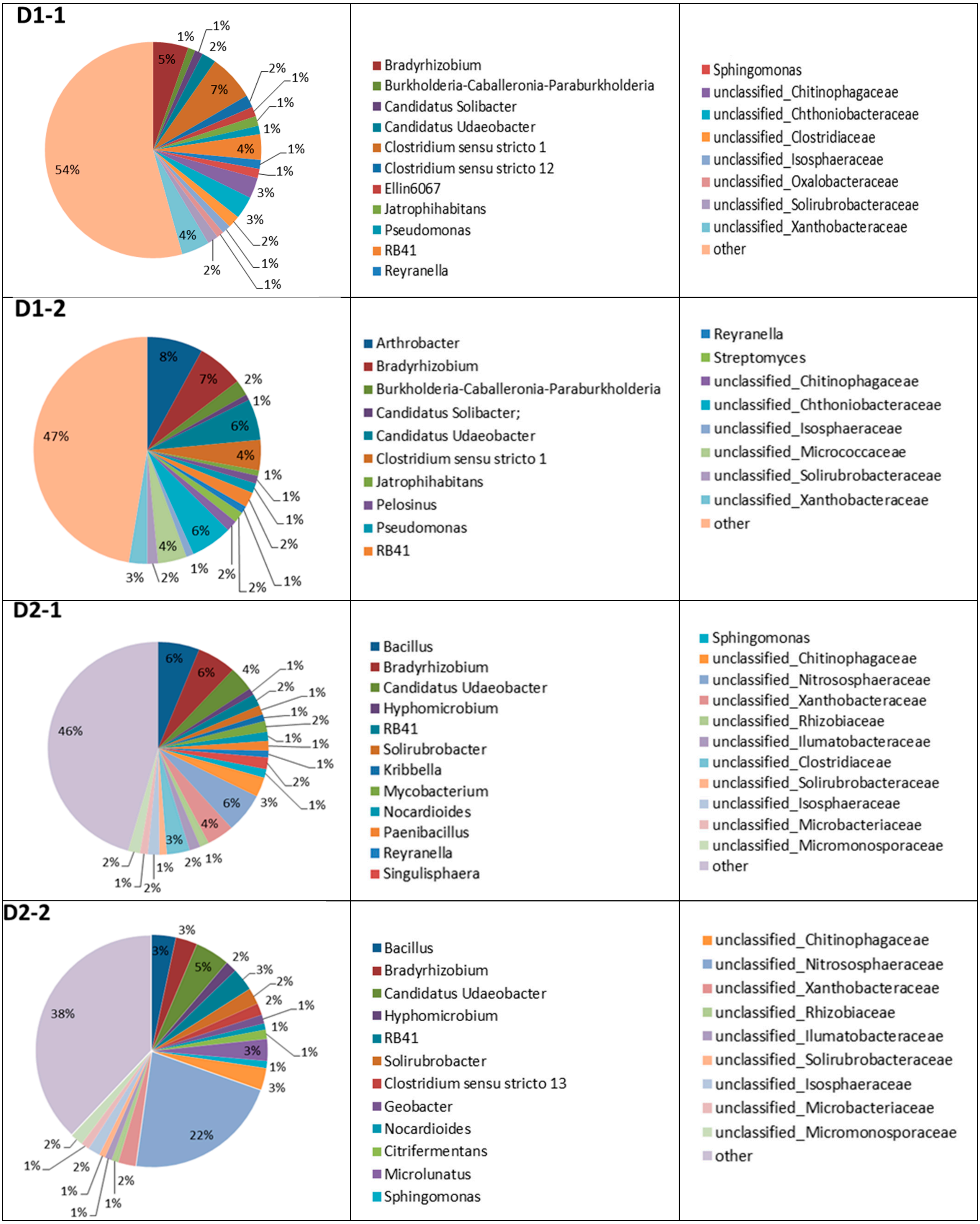

Figure S3 Relative abundance of bacteria and archaea on the genus level in Darkhituy soils (Sites D1 and D2).

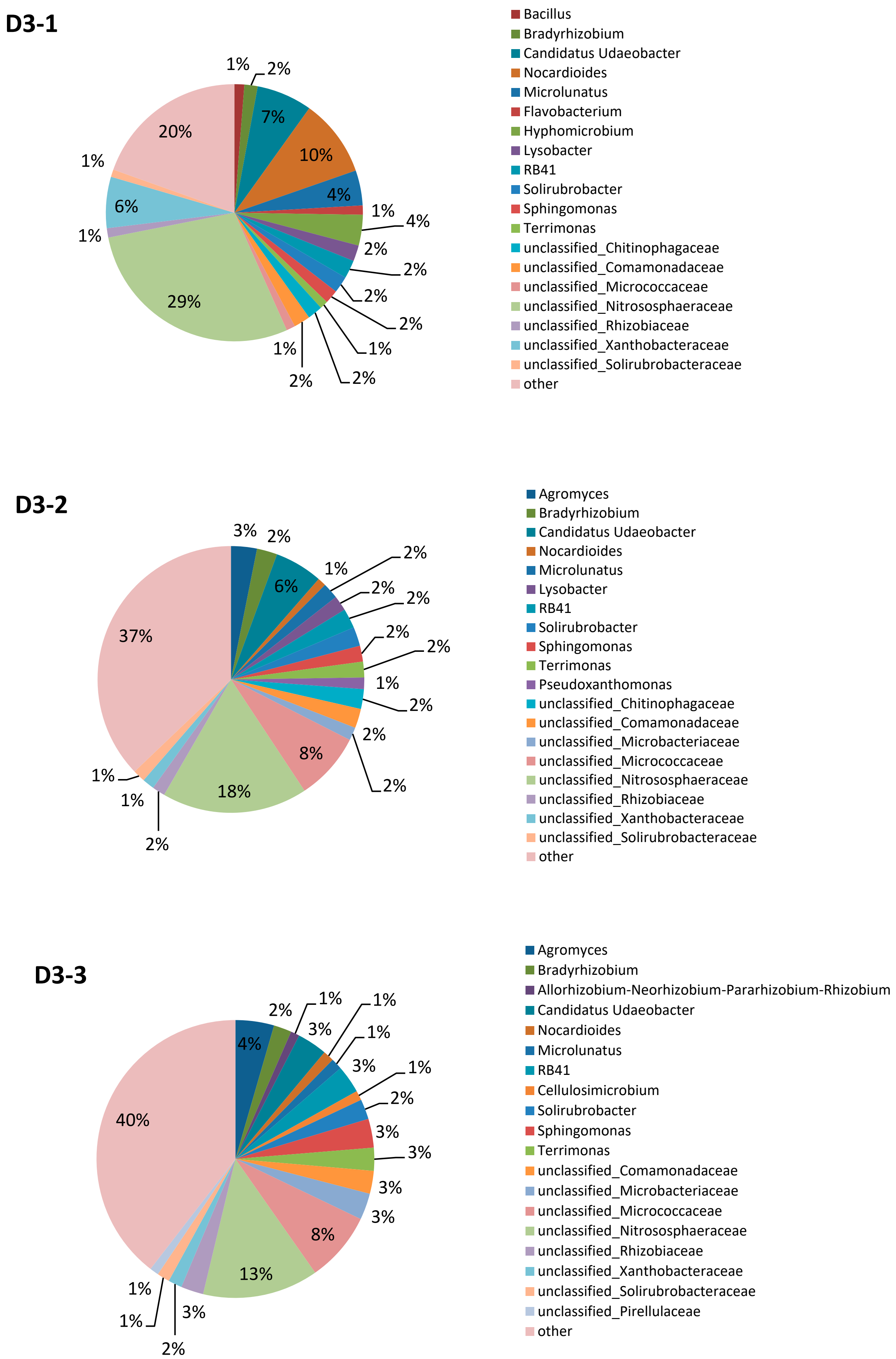

Figure S4 Relative abundance of bacteria and archaea on the genus level in Darkhituy soils (Site D3).

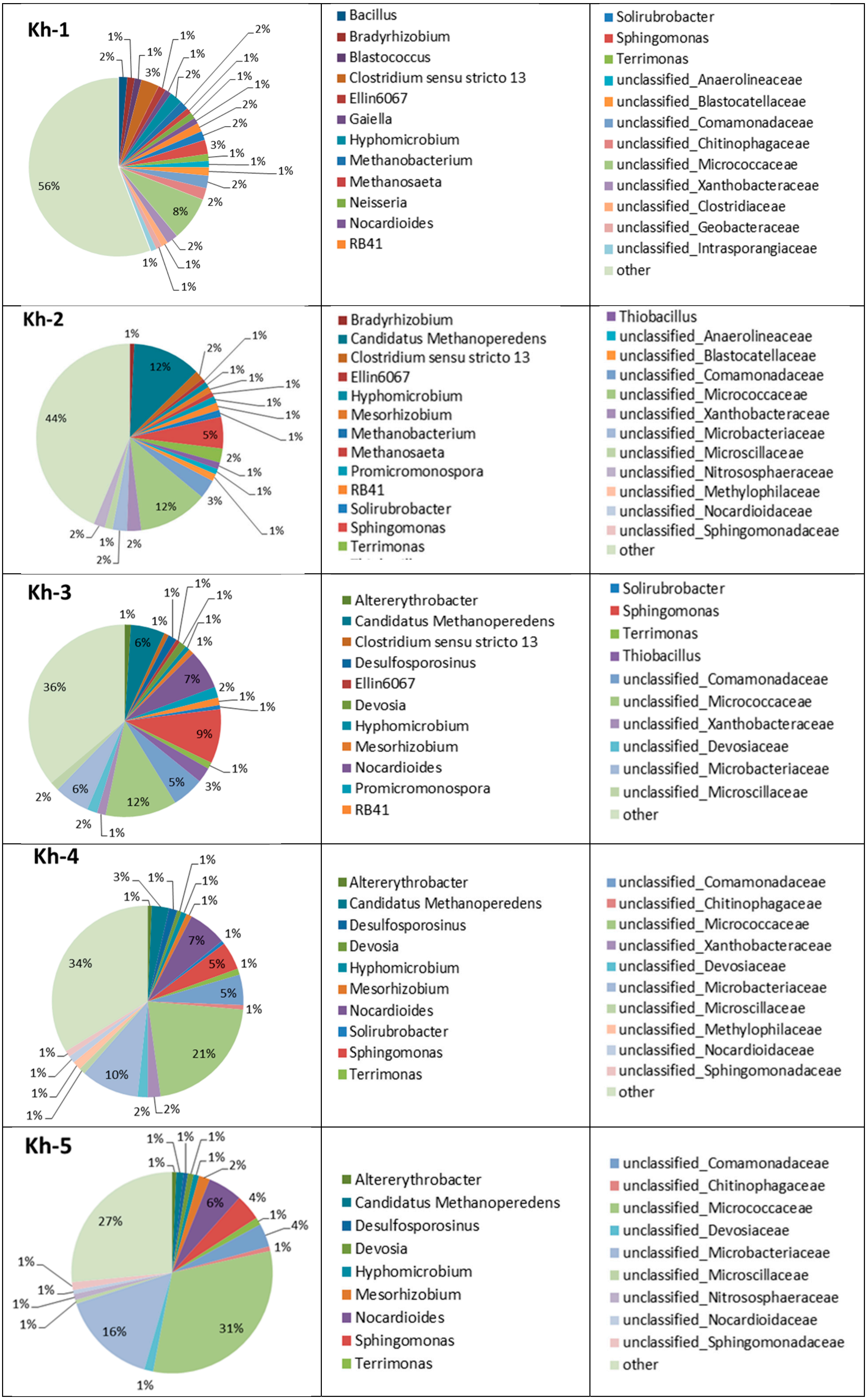

Figure S5 Relative abundance of bacteria and archaea on the genus level in Khaimisan soil.

Table S1 Characteristics of the samples collected from sampling sites of the Darkhituy-Khaimisan transect.

Sampling site	Depth, cm	T, ^{0}C	W, %
D1	at the soil surface	6.4±0.1	-
	0-5	1.6±0.1	110.46
	5-15*	0.1	65.86±14.64
	17-25*; permafrost at 17 cm	-0.5	62.56±12.01
D2	at the soil surface	5.8±1.3	-
	0-5	5.2±1.2	110.19
	5-15*	2.0±0.2	64.43±7.51
	15-26	0.2±0.008	70.04±1.91
	27-37*; permafrost at 27 cm	-0.4	70.61±1.22
D3	at the soil surface	5.7±1.86	-
	0-5	5.1±1.2	39.34±1.2
	5-10*	4.0±1.1	18.35±0.7
	10-20*	2.1±0.4	19.05±0.4
	20-30*	0.9±0.01	19.23±.0.9
	30-40; permafrost at 37 cm	-0.1	19.72±0.7
D4	at the soil surface	14.2	-
	0-10*	9.6±1.5	41.97±9.46
	10-30*	4.1±1.1	23.6±2.9
	30-50*	1.6±0.4	15.36±2.0
	50-70*; permafrost at 67 cm	0.2	32.72
	68-75*; permafrost	-0.2	22.12

“-“ not determined; *samples selected for molecular genetic studies.

Table S2 Relative abundance (%) of microbial phyla in soil community of the Darkhituy-Khaimisan transect

Phylum	D1-1	D1-2	D2-1	D2-2	D3-1	D3-2	D3-3	Kh-1	Kh-2	Kh-3	Kh-4	Kh-5
Crenarchaeota	0.05	0.32	6.38	22.42	29.04	18.48	14.33	1.06	2.29	0.07	0.03	0.94
Halobacterota	0	0	0	0	0	0	0	3.66	14.05	5.86	2.87	1.09
Euryarchaeota	0	0	0	0	0	0	0	1.61	0.35	0	0.15	0
Thermoplasmatota	0	0	0	0	0	0	0	0.37	0.19	0	0	0
Acidobacteriota	10.9	8.21	3.63	5.38	3.74	4	4.93	5.48	5.14	4.83	3.4	2.41
Actinobacteriota	12.64	23.59	16.49	16.6	16.03	28.36	30.99	24.16	23.99	32.46	44.17	55.64
Bacteroidota	5.27	2.17	4.6	4.56	5.03	5.72	6.37	6.68	7.74	4.21	3.88	3.15
Desulfobacterota	0.05	0.27	0.1	3.46	0.27	0.1	0.17	1.92	0.69	0.58	0.32	0.19
Chloroflexi	0	0.02	0	0.06	0.17	0.14	0.1	1.49	0.95	0.29	0.55	0.27
Firmicutes	29.24	17.53	23.34	10.15	2.82	1.17	0.83	11.79	4.37	4.3	2.95	1.87
Gemmatimonadota	1.67	1.18	1.03	1.16	1.38	1.61	1.77	0.36	0.2	0.26	0.035	0.036
Planctomycetota	2.38	1.98	3.98	1.27	0.97	1.17	1.48	1.02	1.12	0.74	0.77	0.41
Proteobacteria; Alphaproteobacteria	19.02	18.65	23.02	17.3	18.615	18.9	19.93	18.44	19.83	23.42	18.7	16.95
Proteobacteria; Gammaproteobacteria	8.28	5.95	6.77	6.4	9.56	10.04	10.17	13.94	14.38	15.49	15.84	11.3
Verrucomicrobiota	6.71	12.15	4.96	6.05	7.77	7.08	4.7	1.17	0.7	1.16	1.15	1.29
Myxococcota	0.19	0.31	0.69	0.62	0.07	0.17	0.001	1.25	0.29	0.26	0.14	0.35
Nitrospirota	0.05	0	0.02	0.14	0.07	0.36	0.5	0.85	0.63	0.51	0.84	1.01
Fibrobacterota	0	0	0	0	0.026	0.04	0.04	0.08	0.1	0.87	0.59	0.24
Patescibacteria	0	0	0	0.04	0.17	0	0	0.05	0.02	0.14	0	0
Bdellovibrionota	0	0	0	0	0.05	0	0	0.04	0	0.09	0	0
Campilobacterota	0	0	0	0	0	0	0	0.13	0	0	0	0
Fusobacteriota	0	0	0	0	0	0	0	0.1	0	0	0	0
Methylomirabilota	0	0	0	0	0	0	0	0.1	0.04	0	0	0
unclassified_*Bacteria*	3.54	5.73	4.65	4.31	3.99	2.6	3.39	4.16	2.68	4.17	3.61	2.75
other	0.01	1.94	0.34	0.08	0.23	0.06	0.3	0.09	0.15	0.3	0	0.11

Table S3 Relationships of microbial community compositions at the phylum level with environmental variables as revealed by Mantel test. Values only significant correlations presented ($P < 0.05$).

Phylum	Depth	T,°C	W, %	pH
Euryarchaeota		0.754		
Halobacterota				0.433
Thermoplasmatota		0.736		
Actinobacteriota	0.799			0.405
Bacteroidota		0.386		
Chloroflexi		0.665		0.544
Firmicutes			0.729	0.400
Fibrobacterota	0.370			0.342
Fusobacteriota		0.660		
Gemmatimonadota	0.376			0.725
Methylomirabilota		0.594		0.366
Nitrospirota	0.393			0.830
Planctomycetota			0.451	0.540
Gammaproteobacteria			0.434	0.763
Verrucomicrobiota				0.748
Campilobacterota		0.695		

Table S4 Correlation matrix for microbial richness and environmental variables as revealed by Spearman Correlation analysis.

	depth	T,°C	W, %	pH	TOC
depth	1				
T,°C	-0,467	1,000			
W, %	-0,301	-0,312	1,000		
pH	0,533	0,422	-0,582	1,000	
TOC	-0,492	0,508	-0,373	-0,168	1,000
ASVs number	-0,398	0,735	0,020	0,313	0,459
Shannon index	-0,775	0,584	0,285	-0,130	0,373
Simpson index	-0,674	0,553	0,407	-0,121	0,284
Nitrososphaeraceae	-0,329	0,073	-0,134	-0,328	0,587
Methanobacteriaceae	-0,327	0,868	0,012	0,453	0,140
Methanocellaceae	-0,333	0,859	-0,041	0,515	0,087
Methanoregulaceae	-0,314	0,834	0,057	0,372	0,180
unclassified_*Methanomicrobiales*	-0,323	0,853	0,040	0,405	0,166
Methanoperedenaceae	0,179	0,125	-0,384	0,592	-0,294
Methanosaetaceae	-0,305	0,797	-0,108	0,563	0,021
Methanosarcinaceae	-0,333	0,871	0,004	0,462	0,134
unclassified_*Methanosarciniales*	-0,090	0,179	-0,207	0,369	-0,177
Methanomassiliicoccaceae	-0,335	0,872	-0,010	0,480	0,120
unclassified_*Methanomassiliicoccales*	-0,090	0,179	-0,207	0,369	-0,177
Acidobacteriaceae (Subgroup 1)	-0,296	-0,314	0,600	-0,611	-0,335
unclassified_*Acidobacteriales*	-0,241	-0,325	0,549	-0,602	-0,394
Solibacteraceae	-0,389	-0,366	0,779	-0,753	-0,297
Bryobacteraceae;	0,868	-0,290	-0,220	0,469	-0,267
Blastocatellaceae	-0,370	0,818	-0,248	0,538	0,148
unclassified_*Blastocatellia*	0,414	0,346	-0,596	0,840	-0,138
Pyrinomonadaceae	-0,411	0,092	0,018	-0,297	0,140
Vicinamibacteraceae	0,296	0,134	-0,623	0,553	-0,050
Thermoanaerobaculacea	-0,125	0,679	-0,162	0,496	0,045
Ilumatobacteracea	-0,329	0,091	0,588	-0,056	-0,166
Micrococcaceae	0,860	-0,064	-0,566	0,763	-0,233
Promicromonosporaceae	0,161	0,146	-0,655	0,497	-0,097
Microbacteriaceae	0,889	-0,307	-0,559	0,530	-0,178
Intrasporangiacea	0,158	0,683	-0,207	0,812	-0,095
Cellulomonadaceae	0,610	-0,154	-0,166	0,578	-0,315
Mycobacteriaceae	-0,605	0,101	0,547	-0,511	0,390
Nocardiaceae	-0,314	0,834	0,057	0,372	0,180
Glycomycetaceae	0,540	-0,144	-0,290	0,435	-0,251
Acidothermaceae	-0,239	-0,185	0,402	-0,421	-0,242
Frankiaceae	-0,308	-0,329	0,615	-0,646	-0,325
Geodermatophilaceae	-0,356	0,858	0,011	0,476	0,128
Sporichthyaceae	-0,197	-0,176	0,315	-0,310	0,256
unclassified_*Frankiales*	-0,461	-0,370	0,745	-0,803	-0,110
Kineosporiaceae	-0,270	-0,210	0,475	-0,369	0,202
Micromonosporaceae	-0,322	-0,320	0,728	-0,636	0,113
Nocardioidaceae	0,501	0,063	-0,609	0,424	0,307
Propionibacteriaceae	-0,250	-0,014	0,036	-0,319	0,421
Pseudonocardiaceae	-0,689	0,590	0,452	-0,212	0,469
Streptomycetaceae	0,112	-0,354	0,267	-0,292	-0,384

Streptosporangiaceae	-0,317	-0,143	0,513	-0,395	0,257
Thermomonosporaceae	-0,264	-0,269	0,507	-0,508	0,147
Actinomycetaceae	-0,314	0,834	0,057	0,372	0,180
Gaiellaceae	-0,519	0,606	0,318	0,028	-0,065
Solirubrobacteraceae	-0,713	0,145	0,161	-0,600	0,458
Rubrobacteriaceae	-0,347	0,079	0,031	-0,361	0,605
Chitinophagaceae	-0,626	0,251	0,092	-0,216	0,199
Flavobacteriaceae	-0,273	0,326	-0,361	-0,134	0,710
Weeksellaceae	-0,194	0,086	-0,016	0,171	-0,030
Crocinitomicaceae	-0,284	0,288	-0,283	-0,175	0,653
Sphingobacteriaceae	-0,112	0,257	-0,415	0,205	0,359
Microscillaceae	0,441	0,186	-0,685	0,798	-0,184
Spirosomaceae	-0,284	0,288	-0,283	-0,175	0,653
Hymenobacteraceae	0,514	-0,058	-0,460	0,628	-0,341
Cyclobacteriaceae	0,834	-0,252	-0,329	0,549	-0,313
unclassified_*Cytophagales*	-0,253	-0,223	0,405	-0,426	0,306
Prevotellaceae	-0,255	0,823	0,010	0,411	0,179
Porphyromonadaceae	-0,163	0,789	0,016	0,456	0,136
Ignavibacteriaceae	0,238	0,577	-0,164	0,674	-0,042
Alicyclobacillaceae	-0,301	-0,191	0,560	-0,505	-0,425
Bacillaceae	-0,341	-0,033	0,567	-0,346	0,257
unclassified_*Bacillales*	-0,362	-0,383	0,729	-0,735	-0,244
Planococcaceae	-0,493	0,382	0,209	-0,239	0,660
Streptococcaceae	-0,196	0,817	-0,054	0,470	0,165
Paenibacillaceae	-0,189	-0,331	0,576	-0,461	0,016
Thermoactinomycetaceae	-0,319	-0,001	0,468	-0,264	-0,412
Staphylococcaceae	-0,009	0,709	-0,194	0,558	0,074
Clostridiaceae	-0,507	-0,017	0,793	-0,504	-0,263
Christensenellaceae	0,209	-0,015	-0,326	0,293	-0,161
Peptostreptococcaceae	-0,197	-0,200	0,640	-0,313	0,019
Anaerovoracaceae	0,045	-0,246	0,470	-0,100	-0,327
Lachnospiraceae	0,072	0,725	-0,114	0,684	-0,001
Thermincolaceae	-0,263	0,820	0,040	0,400	0,167
Sporomusaceae	-0,190	-0,295	0,621	-0,508	-0,484
Veillonellaceae	-0,221	0,802	0,044	0,425	0,148
Symbiobacteraceae	-0,269	-0,231	0,469	-0,512	-0,287
Desulfitobacteriaceae	0,724	-0,123	-0,418	0,626	-0,262
Acetobacteraceae	-0,360	-0,226	0,615	-0,569	-0,287
Inquilinaceae	-0,239	-0,173	0,381	-0,369	0,288
Azospirillaceae	-0,347	0,870	-0,057	0,507	0,117
Caulobacteraceae	0,142	0,450	-0,435	0,570	0,287
Hyphomonadaceae	-0,393	0,730	0,204	0,211	0,285
Dongiaceae	-0,563	0,578	0,415	0,044	0,217
Micropepsaceae	-0,330	-0,368	0,755	-0,627	-0,011
Reyranellaceae	-0,327	-0,436	0,629	-0,801	-0,076
Beijerinckiaceae	-0,625	0,281	0,466	-0,448	0,044
Devosiaceae	0,819	-0,122	-0,585	0,730	-0,268
Hyphomicrobiaceae	-0,385	0,552	-0,203	-0,005	0,690
Rhizobiaceae	-0,128	0,135	-0,482	0,018	0,563
Labraceae	-0,372	0,017	0,167	-0,423	0,629
unclassified_*Rhizobiales*	0,046	0,468	-0,706	0,580	0,313
Xanthobacteraceae	-0,540	-0,310	0,750	-0,889	0,031

Rhodomicrobiaceae	-0,314	0,834	0,057	0,372	0,180
Sphingomonadaceae	0,595	0,061	-0,753	0,758	-0,155
Rhodobacteraceae	-0,015	-0,088	-0,270	-0,089	0,061
Burkholderiaceae	-0,204	-0,355	0,621	-0,604	-0,361
Alcaligenaceae	-0,239	-0,173	0,381	-0,369	0,288
Comamonadaceae	0,624	0,152	-0,769	0,843	-0,094
Hydrogenophilaceae	0,320	0,025	-0,447	0,540	-0,280
Neisseriaceae	-0,253	0,823	0,020	0,409	0,180
Nitrosomonadaceae	-0,087	0,318	0,026	0,471	-0,364
Oxalobacteraceae	0,032	-0,006	0,175	0,153	-0,485
Sutterellaceae	-0,293	0,831	-0,094	0,565	0,105
Methylophilaceae	0,539	-0,171	-0,140	0,353	-0,209
unclassified_*Burkholderiales*	0,728	0,024	-0,337	0,787	-0,402
Diplorickettsiaceae	-0,305	-0,148	0,110	-0,423	0,489
Legionellaceae	-0,185	-0,237	0,372	-0,545	-0,146
Methylomonadaceae	0,580	-0,159	-0,160	0,433	-0,247
Pasteurellaceae	-0,144	0,788	-0,062	0,478	0,175
Pseudomonadaceae	-0,407	-0,361	0,470	-0,825	-0,119
Moraxellaceae	0,086	-0,122	-0,285	-0,030	0,026
Steroidobacteraceae	-0,087	0,381	0,057	0,192	0,470
Rhodanobacteraceae	-0,523	0,656	0,196	0,113	0,388
Xanthomonadaceae	-0,138	0,265	-0,678	-0,021	0,769
unclassified_*Xanthomonadales*	-0,324	-0,269	0,668	-0,544	0,108
Geobacteraceae	-0,090	0,156	0,418	0,131	-0,257
Syntrophobacteraceae	-0,314	0,834	0,057	0,372	0,180
Syntrophorhabdaceae	-0,314	0,834	0,057	0,372	0,180
Desulfocapsaceae	0,480	-0,030	-0,404	0,657	-0,354
Desulfomonilaceae	0,517	-0,132	-0,308	0,434	-0,250
unclassified_*Syntrophales*	0,209	-0,015	-0,326	0,293	-0,161
Gemmatimonadaceae	-0,610	-0,182	0,259	-0,843	0,332
Myxococcaceae	-0,339	0,838	-0,224	0,543	0,129
unclassified_*Myxococcales*	-0,208	0,080	0,361	-0,223	-0,229
Anaeromyxobacteraceae	-0,162	0,211	0,403	0,050	-0,097
Polyangiaceae	-0,232	0,240	0,490	0,017	-0,032
Haliangiaceae	-0,024	0,105	0,418	0,048	0,103
Nannocystaceae	-0,314	0,834	0,057	0,372	0,180
Nitrospiraceae	0,319	0,561	-0,587	0,898	-0,062
Leptospirillaceae	0,876	-0,276	-0,263	0,566	-0,299
Gemmataceae	-0,501	-0,240	0,820	-0,703	-0,142
Isosphaeraceae	-0,297	-0,438	0,763	-0,701	-0,093
Pirellulaceae	-0,438	0,249	-0,312	-0,047	0,490
Rubinisphaeraceae	-0,430	0,711	0,195	0,241	0,266
Chthoniobacteraceae	-0,500	-0,281	0,434	-0,866	0,164
Pedosphaeraceae	-0,294	-0,376	0,541	-0,649	-0,332
Opitutaceae	0,917	-0,228	-0,419	0,704	-0,374
Rubritaleaceae	0,529	0,396	-0,694	0,795	0,111
Verrucomicrobiaceae	-0,090	0,179	-0,207	0,369	-0,177
Ktedonobacteraceae	-0,090	-0,258	0,354	-0,386	-0,313
Anaerolineaceae	0,009	0,750	-0,316	0,796	-0,004
Caldilineaceae	-0,314	0,834	0,057	0,372	0,180
Saccharimonadaceae	-0,146	0,397	-0,380	0,178	0,390
Bacteriovoracaceae	-0,078	0,456	-0,405	0,319	0,249

Fibrobacteraceae	0,610	-0,094	-0,416	0,536	-0,240
Fusobacteriaceae	-0,268	0,651	-0,168	0,535	-0,039
Leptotrichiaceae	-0,141	0,757	0,031	0,458	0,118
Campylobacteraceae	-0,314	0,834	0,057	0,372	0,180
Methylomirabilaceae	-0,137	0,781	-0,052	0,595	0,036
